# Supplementary material for: Activation of NIX-mediated mitophagy by an interferon regulatory factor homologue of human herpesvirus
Source: Nat Commun. 2019 Jul 19;10:3203. doi: 10.1038/s41467-019-11164-2 (PMC6642096; doi:10.1038/s41467-019-11164-2)
Supplement: Supplementary file 3 — Reporting Summary [file 41467_2019_11164_MOESM3_ESM.pdf]

nature research

Corresponding author(s): DBPR-18-37437B

Last updated by author(s): Jun 18, 2019

Reporting Summary

Nature Research wishes to improve the reproducibility of the work that we publish. This form provides structure for consistency and transparency in reporting. For further information on Nature Research policies, see Authors & Referees and the Editorial Policy Checklist .

Statistics

For all statistical analyses, confirm that the following items are present in the figure legend, table legend, main text, or Methods section.

n/a	Confirmed	
☐	☒	The exact sample size (n) for each experimental group/condition, given as a discrete number and unit of measurement
☐	☒	A statement on whether measurements were taken from distinct samples or whether the same sample was measured repeatedly
☐	☒	The statistical test(s) used AND whether they are one- or two-sided<br>*Only common tests should be described solely by name; describe more complex techniques in the Methods section.*
☐	☒	A description of all covariates tested
☒	☐	A description of any assumptions or corrections, such as tests of normality and adjustment for multiple comparisons
☐	☒	A full description of the statistical parameters including central tendency (e.g. means) or other basic estimates (e.g. regression coefficient) AND variation (e.g. standard deviation) or associated estimates of uncertainty (e.g. confidence intervals)
☐	☒	For null hypothesis testing, the test statistic (e.g. F, t, r) with confidence intervals, effect sizes, degrees of freedom and P value noted<br>*Give P values as exact values whenever suitable.*
☒	☐	For Bayesian analysis, information on the choice of priors and Markov chain Monte Carlo settings
☒	☐	For hierarchical and complex designs, identification of the appropriate level for tests and full reporting of outcomes
☒	☐	Estimates of effect sizes (e.g. Cohen's d, Pearson's r), indicating how they were calculated

Our web collection on statistics for biologists contains articles on many of the points above.

Software and code

Policy information about availability of computer code

Data collection	No software was used for data collection.
Data analysis	ImageJ (FIJI), FCS Express 6 Flow, and Prism 8 were used for data analysis and visualization.

For manuscripts utilizing custom algorithms or software that are central to the research but not yet described in published literature, software must be made available to editors/reviewers. We strongly encourage code deposition in a community repository (e.g. GitHub). See the Nature Research guidelines for submitting code & software for further information.

Data

Policy information about availability of data

All manuscripts must include a data availability statement. This statement should provide the following information, where applicable:

- Accession codes, unique identifiers, or web links for publicly available datasets
- A list of figures that have associated raw data
- A description of any restrictions on data availability

All data generated or analyzed during this study are included in this published article and its Source Data files, or are available on request.

Field-specific reporting

Please select the one below that is the best fit for your research. If you are not sure, read the appropriate sections before making your selection.

☒ Life sciences ☐ Behavioural & social sciences ☐ Ecological, evolutionary & environmental sciences

For a reference copy of the document with all sections, see nature.com/documents/nr-reporting-summary-flat.pdf

Life sciences study design

All studies must disclose on these points even when the disclosure is negative.

Sample size	Sample size was determined according to standard practices in the field. All cell-based assays were performed 2 to 3 independent experiments as described in figure legends. No statistical methods were used to predetermine sample sizes. Representative microscopy images were presented from more than 10 randomly-captured images.
Data exclusions	No data were excluded.
Replication	All biochemical and cell-based assays were performed in at least two replicates as described in figure legends. Data from multiple experiments and multiple replicates were analyzed for statistical significance.
Randomization	No randomization was performed.
Blinding	Electron microscopy images were blindly and randomly taken.

Reporting for specific materials, systems and methods

We require information from authors about some types of materials, experimental systems and methods used in many studies. Here, indicate whether each material, system or method listed is relevant to your study. If you are not sure if a list item applies to your research, read the appropriate section before selecting a response.

Materials & experimental systems

n/a	Involved in the study	
☐	☒	Antibodies
☐	☒	Eukaryotic cell lines
☒	☐	Palaeontology
☒	☐	Animals and other organisms
☒	☐	Human research participants
☒	☐	Clinical data

Methods

n/a	Involved in the study	
☒	☐	ChIP-seq
☐	☒	Flow cytometry
☒	☐	MRI-based neuroimaging

Antibodies

Antibodies used

1. Anti-vIRF-1; a gift from Dr. Gary Hayward (Johns Hopkins School of Medicine)
2. Anti-MTCO2; abcam (ab110258)
3. Anti-MTCO2; abcam (ab79393)
4. Anti-NIX; abcam (ab8399)
5. Anti-V5 tag; Bethyl Laboratories (S190-119A)
6. Anti-V5 tag-agarose; Bethyl Laboratories (S190-119)
7. Anti-NIX (D4R4B); Cell Signaling Technology (#12396)
8. Anti-BNIP3; Santa Cruz Biotechnology (sc-56167)
9. Anti-NBR1 (D2E6); Cell Signaling Technology (#9891)
10. Anti-V5 tag (D3H8Q); Cell Signaling Technology (#13202)
11. Anti-DYKDDDDK tag; Cell Signaling Technology (#2368)
12. Anti-PARP (46D11); Cell Signaling Technology (#9532)
13. Anti-DRP1 (D6C7); Cell Signaling Technology (#8570)
14. Anti-HA (7C9); Chromotek GMBH (7C9-100)
15. Anti-FUNDC1; LifeSpan BioSciences (LS-C354368)
16. Anti-p62/SQSTM1; MBL International (PM045)
17. Anti-LC3B; Novus Biologicals (NB100-2220)
18. Anti-beta-Actin; Proteintech (60008-1-lg)
19. Anti-GST (B-14); Santa Cruz Biotechnology (sc-138)
20. Anti-TOM20 (F-10); Santa Cruz Biotechnology (sc-17764)
21. Anti-NIX (E-1); Santa Cruz Biotechnology (sc-166314)
22. Anti-Optineurin (C2); Santa Cruz Biotechnology (sc-166576)
23. Anti-Calcoco/NDP52 (F-6); Santa Cruz Biotechnology (sc-376540)
24. Anti-HSP60 (B-9); Santa Cruz Biotechnology (sc-271215)
25. Anti-ORF45 (2D4A5); Santa Cruz Biotechnology (sc-53883)
26. Anti-K8.1 (4A4); Santa Cruz Biotechnology (sc-65446)
27. Anti-mtTFA/TFAM; Santa Cruz Biotechnology (sc-376672)
28. Anti-Flag tag (M2); Sigma (F3165)

29. Anti-LAMP1; Sino Biological (112150R107)
30. Anti-V5 tag; Thermo Fisher Scientific (R960-25)
31. Anti-HIV-1 TAT; Santa Cruz Biotechnology (sc-65915)
32. Anti-Prohibitin; Santa Cruz Biotechnology (sc-28259)

Validation

1. Anti-vIRF-1;
specific to HHV-8 vIRF-1, validated for immunoblotting (IB) and immunofluorescence staining(IF) (Hwang KY and Choi YB (2016), J. Virol. 90:506; Choi YB et al, (2012), PLoS Pathog. 8:e1002748, Choi YB and Nicholas J (2010), PLoS Pathog. 6:e1001031)
2. Anti-MTCO2; abcam (ab110258)
manufacturer-tested applications: IB, IF, and flow cytometry (FC). We used this antibody for image and flow cytometry analyses.
3. Anti-MTCO2; abcam (ab79393)
manufacturer-tested applications: IB, IF, and FC. We used this antibody in Figs. 1b, 1e, and 2a and supplementary Fig. 1e.
4. Anti-NIX; abcam (ab8399)
manufacturer-tested applications: IB and IF. We used this antibody in Figs. 3a and 3d.
5. Anti-V5 tag; Bethyl Laboratories (S190-119A)
manufacturer-proposed application: IB, immunoprecipitation (IP), and IF. 24 product citations. We used this antibody for IF of V5-tagged NIX.
6. Anti-V5 tag-agarose; Bethyl Laboratories (S190-119)
manufacturer-tested and paper-cited application: IP. We used this antibody for IP of V5-tagged NIX (Fig. 5h).
7. Anti-NIX (D4R4B); Cell Signaling Technology (#12396)
manufacturer-tested application: IB. 14 product citations. We used this antibody for IB (Fig. 3e).
8. Anti-BNIP3; Santa Cruz Biotechnology (sc-56167)
manufacturer-tested applications: IB, IP, and IF. 24 product citations. We used this antibody for IB (Supplementary Fig. 2).
9. Anti-NBR1 (D2E6); Cell Signaling Technology (#9891)
manufacturer-tested application: IB. 10 product citations. We used this antibody for IB (Fig. 3a).
10. Anti-V5 tag (D3H8Q); Cell Signaling Technology (#13202)
manufacturer-tested application: IB, IP, and IF. 28 product citations. We used this antibody for IF (supplementary Fig. 6).
11. Anti-DYKDDDDK tag; Cell Signaling Technology (#2368)
manufacturer-proposed applications: IB, IP, and FC. 288 product citations. We used this antibody for IF of Flag-tagged vIRF-1.
12. Anti-PARP (46D11); Cell Signaling Technology (#9532)
manufacturer-tested application: IB. 440 product citations. We used this antibody for IB (Figs. 1e, 6j, and 6k).
13. Anti-DRP1 (D6C7); Cell Signaling Technology (#8570)
manufacturer-tested application: IB. 56 product citations. We used this antibody for IB (Fig. 3a).
14. Anti-HA (7C9); Chromotek GMBH (7C9-100)
manufacturer-tested applications: IB and IF. 288 product citations. We used this antibody for IB (Fig. 5h) and IF (Fig. 7h).
15. Anti-FUNDC1; LifeSpan BioSciences (LS-C354368)
manufacturer-tested application: IB. We used this antibody for IB (Fig. 3a).
16. Anti-p62/SQSTM1; MBL International (PM045)
manufacturer-tested applications: IB, IP, and IF. 22 product citations. We used this antibody for IB (Fig. 3a).
17. Anti-LC3B; Novus Biologicals (NB100-2220)
manufacturer-validated by Biological and Genetic Strategies. 504 product citations for IB. We used this antibody for IB (Figs. 3a and 3c).
18. Anti-beta-Actin; Proteintech (60008-1-lg)
manufacturer-tested applications: IB, IF, FC, and IF. 1220 product citations. We used this antibody for IB.
19. Anti-GST (B-14); Santa Cruz Biotechnology (sc-138)
manufacturer-tested application: IB. 730 product citations. We used this antibody for IB (Figs. 4e and 4f).
20. Anti-TOM20 (F-10); Santa Cruz Biotechnology (sc-17764)
manufacturer-tested applications: IB and IF. 142 product citations. We used this antibody for IF and IB.
21. Anti-NIX (E-1); Santa Cruz Biotechnology (sc-166314)
manufacturer-proposed applications: IB, IP, and IF. We used this antibody for IP (Fig. 4a) and PLA (Fig. 4d).
22. Anti-Optineurin (C2); Santa Cruz Biotechnology (sc-166576)
manufacturer-tested applications: IB, IP, and IF. 9 product citations. We used this antibody for IB (Fig. 3a).
23. Anti-Calcoco/NDP52 (F-6); Santa Cruz Biotechnology (sc-376540)
manufacturer-tested applications: IB, IP, and IF. 3 product citations. We used this antibody for IB (Fig. 3a).
24. Anti-HSP60 (B-9); Santa Cruz Biotechnology (sc-271215)
manufacturer-tested applications: IB, IP, and IF. 4 product citations. We used this antibody for IB (Figs. 3a, 3c, and 3d).
25. Anti-ORF45 (2D4A5); Santa Cruz Biotechnology (sc-53883)
manufacturer-proposed applications: IB. 7 product citations. We used this antibody for IB (Fig. 8b).
26. Anti-K8.1 (4A4); Santa Cruz Biotechnology (sc-65446)
manufacturer-proposed applications: IB. 3 product citations. We used this antibody for IB (Fig. 8b).
27. Anti-mtTFA/TFAM; Santa Cruz Biotechnology (sc-376672)
manufacturer-tested applications: IB, IP, and IF. 3 product citations. We used this antibody for IF of mitochondria (Figs. 3g, 4h, 5g, and 7c) and IB (Fig. 2d).
28. Anti-Flag tag (M2); Sigma (F3165)
manufacturer-tested applications: IB, IP, and IF. We used this antibody for IB (Figs. 4g, 5d, and 5f; supplementary Figs. 3 and 5b).
29. Anti-LAMP1; Sino Biological (11215-R107)

manufacturer-tested applications: IB, IP, ELISA, and IF. >250 product citations. We used this antibody for IF (Figs. 6d and 6f).
30. Anti-V5 tag; Thermo Fisher Scientific (R960-25)
manufacturer-tested applications: IB and IF. 484 product citations. We used this antibody for IB (Figs. 5d, 5f, and 5h, supplementary Figs. 3 and 5b.
31. Anti-HIV-1 TAT; Santa Cruz Biotechnology (sc-65915)
manufacturer-tested applications: IB and IP. 1 product citation. We used this antibody for IF (Fig. 7b).
32. Anti-Prohibitin; Santa Cruz Biotechnology (sc-28259)
14 product citations. We used this antibody for IF (Fig. 7b).

Eukaryotic cell lines

Policy information about cell lines

Cell line source(s)	293T and iBCBL-1 from ATCC; HeLa.Kyoto from Dr. Ron R. Kopito; iSLK from Dr. Jae U. Jung
Authentication	validated by the providers. iBCBL-1 and iSLK cells were verified by immunoblotting analysis of doxycycline-inducible RTA expression.
Mycoplasma contamination	We confirmed to be negative for mycoplasma and used Plasmocin Prophylatic (InvivoGen) to prevent mycoplasma contamination.
Commonly misidentified lines (See ICLAC register)	not included in this study.

Flow Cytometry

Plots

Confirm that:

- [x] The axis labels state the marker and fluorochrome used (e.g. CD4-FITC).
- [x] The axis scales are clearly visible. Include numbers along axes only for bottom left plot of group (a 'group' is an analysis of identical markers).
- [ ] All plots are contour plots with outliers or pseudocolor plots.
- [x] A numerical value for number of cells or percentage (with statistics) is provided.

Methodology

Sample preparation	Cells were fixed in 4% formaldehyde at 37℃ for 10 min and permeabilized in a final concentration of 90% methanol. The fixed cells were washed twice with blocking buffer (0.5% BSA in PBS) by centrifugation at 3,000 x g for 5 min and incubated with the indicated primary antibody in blocking buffer for 1 h at room temperature. Cells were washed twice with blocking buffer and stained with Alexa Flour® 488-conjugated goat anti-mouse IgG antibody in blocking buffer.<br><br>For Apoptosis analysis, cells were washed in PBS and Annexin-V binding buffer, and incubated in Annexin-V-Alexa Fluor 647 ffor 15 min.
Instrument	FACSCalibur (Becton Dickinson) and Cellometer Vision CBA (Nexcelom)
Software	CellQuest and Nexcelom were used for data acquisition and FCS express 6 flow was used for data analysis.
Cell population abundance	We determined mitochondrial content of whole cell population.
Gating strategy	No gating applied.

- [ ] Tick this box to confirm that a figure exemplifying the gating strategy is provided in the Supplementary Information.
